# Development and validation of a novel model to predict post-stroke cognitive impairment within 6 months after acute ischemic stroke

**DOI:** 10.3389/fneur.2024.1451786

**Published:** 2024-12-24

**Authors:** Ming Wei, Xiaofeng Zhu, Xiu Yang, Jin Shang, Qiang Tong, Qiu Han

**Affiliations:** Department of Neurology, Huai’an First People’s Hospital, The Affiliated Huai’an No.1 People’s Hospital of Nanjing Medical University, Huai’an, Jiangsu, China

**Keywords:** clinical prediction model, acute ischemic stroke, post-stroke cognitive impairment, atrial fibrillation, cognitively normal

## Abstract

**Background:**

Cognitive decline following acute ischemic stroke (AIS), termed post-stroke cognitive impairment (PSCI), is a prevalent phenomenon that significantly elevates disability and mortality rates among affected patients. The objective of this investigation was to develop a robust clinical prediction model capable of forecasting PSCI within six months post-AIS and subsequently validate its effectiveness.

**Methods:**

A cohort of 573 AIS patients was stratified into two groups: those with PSCI (260 cases) and those who remained cognitively normal (CN) (313 cases). These patients were further subdivided into three distinct cohorts: a development cohort comprising 193 AIS patients, an internal validation cohort with 193 AIS patients, and an external validation cohort encompassing 187 AIS patients. A thorough multifactor logistic regression analysis was conducted to identify independent predictors of PSCI, which were subsequently incorporated into the prediction model for comprehensive analysis and validation. The discriminatory power, calibration accuracy, and clinical net benefits of the prediction model were rigorously evaluated using the area under the receiver operating characteristic curve (AUC-ROC), calibration plots, and decision curve analyses, respectively.

**Results:**

Utilizing a meticulously selected panel of variables, including smoking status, alcohol consumption, female gender, low educational attainment, NIHSS score at admission, stroke progression, diabetes mellitus, atrial fibrillation, stroke localization, HCY levels, and Lp-PLA2 levels, a clinical prediction model was formulated to predict the occurrence of PSCI within six months of AIS. The model demonstrated AUC-ROC values of 0.898 (95%CI, 0.853–0.942), 0.847 (95%CI, 0.794–0.901), and 0.849 (95%CI, 0.7946–0.9031) in the development, internal validation, and external validation cohorts, respectively. Further validation through calibration curve analyses, Hosmer-Lemeshow goodness-of-fit tests, and additional metrics confirmed the model’s impressive predictive performance.

**Conclusion:**

The proposed model exhibits strong discriminative ability for predicting PSCI and holds considerable promise for guiding clinical decision-making. However, ongoing optimization with multicenter data is necessary to bolster its robustness and broaden its applicability.

## 1 Introduction

Post-stroke cognitive impairment denotes cognitive deficiencies emerging subsequent to a cerebrovascular accident, devoid of discernible pre-stroke cognitive deterioration (1). This condition is prevalent among adults and correlates with heightened disability and mortality rates among stroke survivors (2, 3). The incidence of PSCI spans a broad spectrum, reported to vary between 20 and 82% (4–6). Considering the neuroplastic potential and neural regenerative capacity within the initial post-stroke months, PSCI may exhibit reversal or amelioration in certain patients (1, 7), however, such enhancements become less evident beyond the six-month mark (8, 9). Consequently, prompt identification of stroke survivors at high risk for PSCI, coupled with timely preventive and therapeutic interventions, is pivotal in mitigating or postponing the onset of PSCI (1–3). This strategy aims to elucidate the risk factors associated with PSCI, thereby enhancing clinical management and fostering the development of tailored interventions. By capitalizing on the unique characteristics of acute ischemic stroke, we endeavor to devise a predictive model tailored to the specific requirements of the Chinese populace, while facilitating early identification of individuals at heightened risk. This proactive methodology holds the promise of substantially influencing patient outcomes and optimizing the overall management of PSCI in post-stroke patients.

## 2 Materials and methods

### 2.1 General information

This research constitutes a retrospective cohort analysis, encompassing clinical data from patients with acute ischemic stroke from August 2019 to October 2022. Prior to disease onset, all patients exhibited normal cognitive function and underwent follow-up assessments at 1, 3, and 6 months post-discharge. Patient follow-up in this context primarily involved reviewing clinical records to ascertain the outcomes of interest over time. Follow-up was conducted according to the local standard of care, which typically involves scheduled visits, lab tests, and imaging studies as deemed necessary by the treating physicians. The frequency and nature of these follow-ups varied based on individual patient needs and clinical protocols in place at the time. Regarding the completion of follow-ups, we meticulously reviewed all available records to determine the number of patients who completed all scheduled visits and those who missed some. Regarding subject exclusion based on data availability for follow-ups, we implemented a rigorous selection criterion to ensure that our sample was representative and unbiased. Specifically, we excluded patients only if they had insufficient data to make a meaningful contribution to the analysis, such as those with no follow-up records at all or those with critical missing data points that could not be reasonably estimated or imputed. However, this exclusion was minimal and did not significantly affect the generalizability of our findings.

Cognitive status was evaluated using instruments such as the Mini-Mental State Examination (MMSE), Montreal Cognitive Assessment (MoCA), and other cognitive rating scales during these follow-up intervals. In the realm of poststroke cognitive impairment screening, the Montreal Cognitive Assessment (MoCA) has been recommended by both the National Institute of Neurological Disorders and Stroke and the Canadian Stroke Network (10). Recent scholarly evaluations have highlighted the MoCA’s superior sensitivity in detecting such impairments, notably absent of a ceiling effect, positioning it as the preeminent tool in this context (11–13). A baseline MoCA score below 22 was deemed indicative of acute cognitive impairment, and patients maintaining a MoCA score under 26 at 6 months were diagnosed with post-stroke cognitive impairment (14–16). Patients with an education duration of less than 12 years were classified as having a low educational level and received a one-point adjustment in their MoCA score accordingly (14). The calculations determined the proportion and quantity of individuals with acute cognitive decline who attained normative cognitive functioning (MoCA ≥26) six months post-AIS. Based on MoCA scores, patients were stratified into the PSCI and CN groups. The data gathered encompassed demographics such as age, gender, education level, height, weight, blood pressure, alcohol consumption, smoking history, and the presence of comorbidities including hypertension, diabetes mellitus (DM), atrial fibrillation (AF), coronary heart disease (CHD), peripheral vascular disease (PVD), stroke localization (Left or right cerebral hemisphere), stroke progression (defined as neurological deterioration occurring within 6 h to 7 days post-AIS, with an increase in NIHSS score by at least 2 points, including 1 point for consciousness and 1 point for limb movement, or a total increase of 4 points), and whether undergone reperfusion therapy. Additionally, admission NIHSS scores were recorded. Furthermore, blood and biochemical indicators such as low-density lipoprotein cholesterol (LDL-C), triglycerides (TG), total cholesterol (TC), beta2-microglobulin, homocysteine (HCY), lipoprotein-associated phospholipase A2 (Lp-PLA2), and lipoprotein A were also collected. This study received approval from the Ethics Committee of the Huai’an First People’s Hospital (Ethics No. KY-2023-076-01).

### 2.2 Inclusion and exclusion criteria

Inclusion criteria:

1. Meeting the diagnostic criteria for acute ischemic stroke;
2. Age over 18 years;
3. Admission within 72 h of onset;
4. Complete clinical and follow-up data;
5. Baseline MoCA score less than 22, and MoCA score still less than 26 at 6 months.

Exclusion criteria:

1. Patients with epileptic seizures, transient ischemic attacks (TIA), or conditions mimicking stroke;
2. Pre-existing cognitive impairment;
3. Patients with mental or psychological disorders;
4. Reversal or normalization of cognitive impairment within 6 months;
5. Acute ischemic stroke with other severe complications (such as heart failure, respiratory failure, liver or kidney failure, severe gastrointestinal bleeding, severe infections, etc.).

### 2.3 Development cohort, internal validation cohort, and external validation cohort

**Figure 1:**
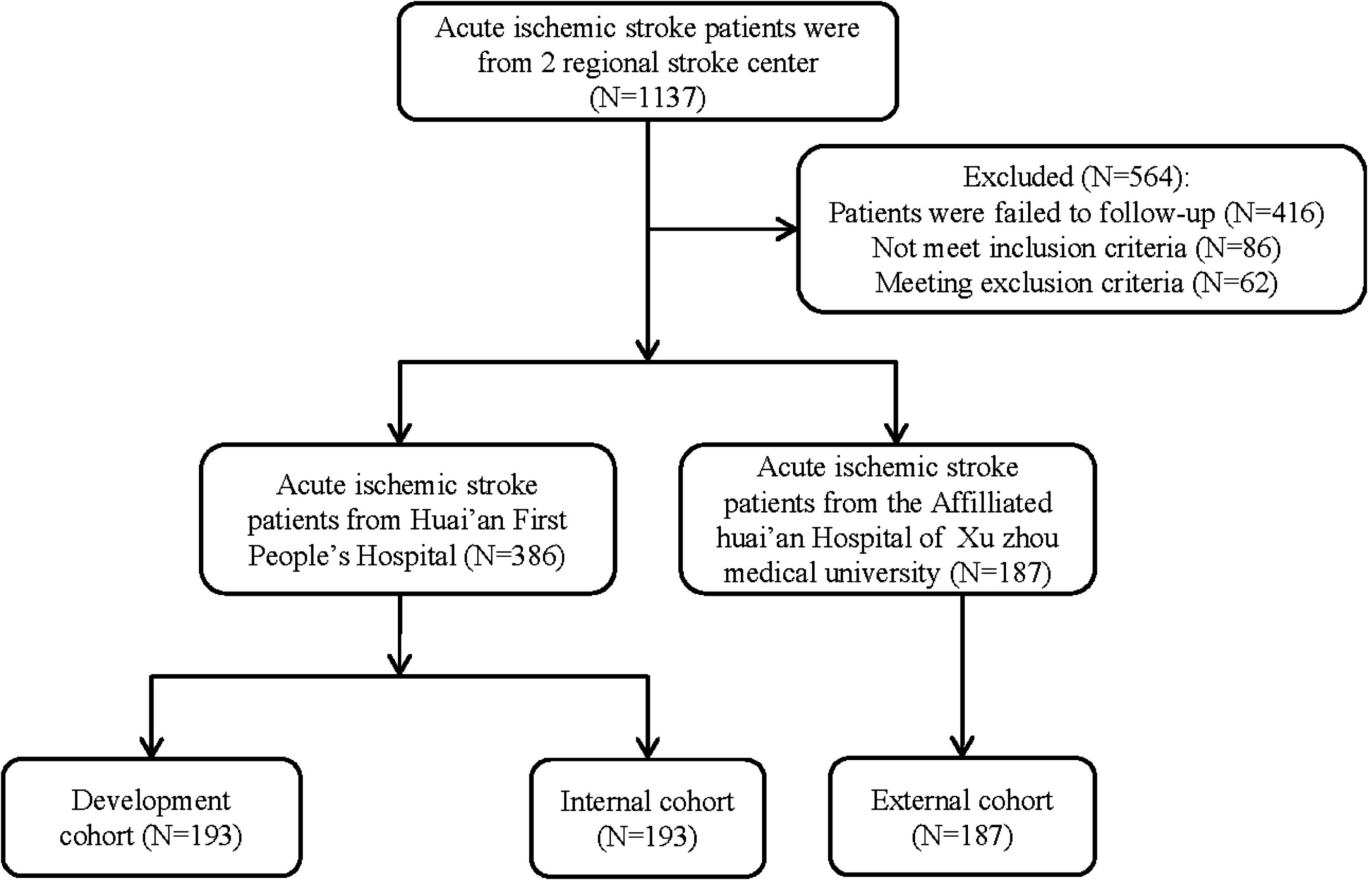
Study flowchart.

A stratified random sampling approach was employed to allocate 386 patients with AIS, admitted to the Stroke Center of the Huai’an First People’s Hospital, into two equal-sized cohorts: a development cohort and an internal validation cohort, each comprising 193 patients. Additionally, based on identical inclusion and exclusion criteria, 187 AIS patients from the Stroke Center of the Affiliated Huai’an Hospital of Xuzhou Medical University were designated as the external validation cohort. Consequently, the study encompassed a total of three cohorts: 193 patients in the development cohort, 193 in the internal validation cohort, and 187 in the external validation cohort (Figure 1).

### 2.4 Statistical analysis

To address missing data in our analysis, we employed multiple imputation techniques, which are widely recognized statistical methods for handling missing data. These techniques involve creating multiple plausible sets of missing values and analyzing each set separately, ultimately combining the results to obtain a single estimate. This approach helps to minimize the bias and variance that can arise from simply excluding patients with missing data. Categorical variables were assessed utilizing the chi-square (χ^2^) test for independence. For continuous variables adhering to normal distribution, the results were reported as the mean accompanied by the standard deviation (SD), with group comparisons conducted through the application of Student’s t-test. Conversely, for variables exhibiting non-normal distribution, the median and interquartile range (IQR) were employed to present the results, and group comparisons were executed using the Wilcoxon rank-sum test. A bidirectional stepwise approach was adopted in the multivariable logistic regression analysis to select variables and construct a clinical prediction model. Variables were selected for inclusion in the clinical prediction model based on their statistical significance in the multivariate logistic regression analysis. Specifically, we used a stepwise variable selection process, which involves entering variables into the model one by one, testing their significance, and removing those that do not contribute significantly to the model. This process was repeated until only the most significant variables remained in the final model. We also considered clinical relevance and previous research when selecting variables for inclusion in the model. For example, we included variables such as age, sex, and vascular risk factors, which are known to be associated with PSCI. Visualization of the model was facilitated by the RMS package within R software, version 4.2.2. The predictive capabilities of the model were evaluated based on its discrimination, calibration, and generalizability, encompassing accuracy and stability. Discrimination was quantified using the area under the receiver operating characteristic (ROC) curve. Additionally, calibration was assessed through calibration curve analysis and the Hosmer-Lemeshow goodness-of-fit test. Both internal and external validations of the model were conducted, utilizing 10-fold cross-validation and the bootstrap method, respectively. All statistical analyses and data visualizations were executed using R software, version 4.2.2. Statistical significance was determined at a *p*-value threshold of <0.05.

## 3 Results

### 3.1 Comparison of demographic and baseline data

This study included 573 patients with AIS from two stroke center. General demographic and baseline characteristics of the total population, PSCI group, and CN group are presented in Table 1. There were statistically significant differences (*p* < 0.05) between the two groups in terms of NIHSS score at admission, HCY, β2. Microglobulin, Lp-PLA2, stroke progression, hypertension, diabetes mellitus, smoking, alcohol consumption, atrial fibrillation, education level, and stroke localization (Table 1).

**Table 1:** Comparison of demographic and baseline data of the all patients from two stroke center.

Variables	Level	Overall	CN	PSCI	*p*
		*N* = 573	*N* = 313	*N* = 260	
NIHSS (median [IQR])		11.00 [6.00, 14.00]	10.00 [6.00, 14.00]	12.00 [8.00, 14.00]	0.003
HCY (median [IQR])		22.75 [12.58, 30.65]	21.19 [10.20, 30.29]	23.74 [15.14, 31.44]	0.001
*β*2.Microglobulin [mean (SD)]		2.47 (0.72)	2.40 (0.76)	2.56 (0.66)	0.006
Lp.PLA2 (median [IQR])		166.16 [133.54, 201.47]	154.58 [124.08, 193.20]	174.88 [144.75, 206.68]	<0.001
BMI [mean (SD)]		25.43 (3.48)	25.44 (3.43)	25.42 (3.54)	0.955
LDL.C [mean (SD)]		2.71 (0.80)	2.72 (0.83)	2.68 (0.78)	0.551
TG [mean (SD)]		4.11 (2.11)	3.96 (2.10)	4.29 (2.11)	0.061
TC [mean (SD)]		3.90 (0.82)	3.91 (0.84)	3.90 (0.79)	0.891
Lipoprotein.A [mean (SD)]		280.57 (32.99)	280.01 (33.45)	281.24 (32.48)	0.657
Age [mean (SD)]		60.16 (11.56)	60.63 (11.88)	59.61 (11.15)	0.294
Stroke progressive (%)	No	472 (82.4)	270 (86.3)	202 (77.7)	0.01
	Yes	101 (17.6)	43 (13.7)	58 (22.3)	
Hypertension (%)	No	275 (48.0)	167 (53.4)	108 (41.5)	0.006
	Yes	298 (52.0)	146 (46.6)	152 (58.5)	
DM (%)	No	263 (45.9)	163 (52.1)	100 (38.5)	0.002
	Yes	310 (54.1)	150 (47.9)	160 (61.5)	
Smoke (%)	No	353 (61.6)	216 (69.0)	137 (52.7)	<0.001
	Yes	220 (38.4)	97 (31.0)	123 (47.3)	
Alcohol (%)	No	375 (65.4)	221 (70.6)	154 (59.2)	0.006
	Yes	198 (34.6)	92 (29.4)	106 (40.8)	
AF (%)	No	522 (91.1)	296 (94.6)	226 (86.9)	0.002
	Yes	51 (8.9)	17 (5.4)	34 (13.1)	
Education (%)	High	349 (60.9)	230 (73.5)	119 (45.8)	<0.001
	Low	224 (39.1)	83 (26.5)	141 (54.2)	
CHD (%)	No	444 (77.5)	237 (75.7)	207 (79.6)	0.312
	Yes	129 (22.5)	76 (24.3)	53 (20.4)	
PVD (%)	No	442 (77.1)	237 (75.7)	205 (78.8)	0.431
	Yes	131 (22.9)	76 (24.3)	55 (21.2)	
Gender (%)	Female	268 (46.8)	142 (45.4)	126 (48.5)	0.513
	Male	305 (53.2)	171 (54.6)	134 (51.5)	
Stroke localization (%)	Left	252 (44.0)	93 (29.7)	159 (61.2)	<0.001
	Right	321 (56.0)	220 (70.3)	101 (38.8)	
Reperfusion therapy (%)	No	457 (79.8)	240 (76.7)	217 (83.5.)	0.056
	Yes	116 (20.2)	73 (23.3)	43 (16.5)	

NIHSS, National Institutes of Health Stroke Scale; DM, diabetes mellitus; AF, atrial fibrillation; CHD, coronary heart disease; PVD, peripheral vascular disease.

A cohort of 386 individuals suffering from acute ischemic stroke was enrolled at the Stroke Center of the Huai’an First People’s Hospital. Demographic details and baseline features of the entire cohort, as well as those belonging to the PSCI and CN groups, are outlined in Table 2. Inspection of Table 2 reveals statistically noteworthy disparities (*p* < 0.05) across the two groups concerning admission NIHSS scores, HCY levels, Lp-PLA2 concentrations, stroke progression rates, hypertension status, diabetes prevalence, atrial fibrillation, smoking habits, alcohol intake, educational attainment, stroke localization, and reperfusion therapy.

**Table 2:** Comparison of demographic and baseline data of the patients from the stroke center of the Huai’an First People’s Hospital.

Variables		Overall	CN	PSCI	*p*
		*N* = 386	*N* = 212	*N* = 174	
NIHSS (median [IQR])		11.00 [6.00, 14.00]	9.50 [5.00, 14.00]	12.00 [8.00, 14.00]	0.012
HCY (median [IQR])		22.72 [12.70, 30.42]	21.01 [10.51, 30.30]	23.58 [15.24, 30.70]	0.02
β2.Microglobulin (mean (SD))		2.48 (0.73)	2.42 (0.78)	2.55 (0.66)	0.081
Lp.pla2 (median [IQR])		167.42 [133.85, 203.85]	156.64 [124.01, 193.66]	176.79 [149.40, 208.25]	<0.001
BMI [mean (SD)]		25.42 (3.48)	25.52 (3.42)	25.30 (3.55)	0.546
LDL.C [mean (SD)]		2.70 (0.80)	2.73 (0.82)	2.66 (0.77)	0.424
TC [mean (SD)]		4.08 (2.14)	3.91 (2.13)	4.29 (2.15)	0.084
TG [mean (SD)]		3.90 (0.81)	3.92 (0.84)	3.87 (0.78)	0.585
Lipoprotein.A [mean (SD)]		281.36 (32.82)	280.48 (33.42)	282.44 (32.14)	0.56
Age [mean (SD)]		60.35 (11.46)	60.83 (11.77)	59.78 (11.08)	0.369
Progressive (%)	No	319 (82.6)	184 (86.8)	135 (77.6)	0.025
	Yes	67 (17.4)	28 (13.2)	39 (22.4)	
Hypertension (%)	No	186 (48.2)	113 (53.3)	73 (42.0)	0.034
	Yes	200 (51.8)	99 (46.7)	101 (58.0)	
DM (%)	No	173 (44.8)	106 (50.0)	67 (38.5)	0.031
	Yes	213 (55.2)	106 (50.0)	107 (61.5)	
Smoke (%)	No	236 (61.1)	145 (68.4)	91 (52.3)	0.002
	Yes	150 (38.9)	67 (31.6)	83 (47.7)	
Alcohol (%)	No	253 (65.5)	150 (70.8)	103 (59.2)	0.023
	Yes	133 (34.5)	62 (29.2)	71 (40.8)	
AF (%)	No	349 (90.4)	199 (93.9)	150 (86.2)	0.018
	Yes	37 (9.6)	13 (6.1)	24 (13.8)	
Education (%)	High	238 (61.7)	157 (74.1)	81 (46.6)	<0.001
	Low	148 (38.3)	55 (25.9)	93 (53.4)	
CHD (%)	No	294 (76.2)	157 (74.1)	137 (78.7)	0.34
	Yes	92 (23.8)	55 (25.9)	37 (21.3)	
PVD (%)	No	292 (75.6)	157 (74.1)	135 (77.6)	0.494
	Yes	94 (24.4)	55 (25.9)	39 (22.4)	
Gender (%)	Female	180 (46.6)	95 (44.8)	85 (48.9)	0.491
	Male	206 (53.4)	117 (55.2)	89 (51.1)	
Stroke localization (%)	Left	181 (46.9)	62 (29.2)	119 (68.4)	<0.001
	Right	205 (53.1)	150 (70.8)	55 (31.6)	
Reperfusion therapy (%)	No	303 (78.5)	157 (74.1)	146 (83.9)	0.026
	Yes	83 (21.5)	55 (25.9)	28 (16.1)	

NIHSS, National Institutes of Health Stroke Scale; DM, diabetes mellitus; AF, atrial fibrillation; CHD, coronary heart disease; PVD, peripheral vascular disease.

An external validation cohort comprising 187 patients with acute ischemic stroke, sourced from the Stroke Center of the Affiliated Huai’an Hospital of Xuzhou Medical University, underwent external scrutiny. The comprehensive demographic profile and baseline attributes of the entire cohort, along with those of the PSCI and CN groups, are detailed in Table 3. A statistical analysis revealed significant disparities (*p* < 0.05) between the two groups concerning HCY levels, β2-microglobulin concentrations, Lp-PLA2 levels, diabetes mellitus presence, smoking habits, educational attainment, and stroke localization as summarized in Table 3.

**Table 3:** Comparison of demographic and baseline data of the patients from the stroke center of the Affiliated Huai’an Hospital of Xuzhou Medical University.

Variables		Overall	CN	PSCI	*p*
		*N* = 187	*N* = 101	*N* = 86	
NIHSS (median [IQR])		11.00 [6.00, 14.00]	10.00 [6.00, 14.00]	12.00 [8.00, 14.75]	0.098
HCY (median [IQR])		23.19 [12.36, 31.46]	22.46 [9.86, 29.88]	24.81 [14.69, 33.55]	0.022
Microglobulin [mean (SD)]		2.46 (0.71)	2.35 (0.73)	2.59 (0.66)	0.022
Lp.pla2 (median [IQR])		165.96 [132.17, 195.18]	153.37 [124.08, 183.37]	173.33 [142.51, 203.92]	0.01
BMI [mean (SD)]		25.46 (3.49)	25.28 (3.45)	25.67 (3.54)	0.445
LDL.C [mean (SD)]		2.72 (0.82)	2.72 (0.84)	2.73 (0.80)	0.929
TG [mean (SD)]		4.17 (2.03)	4.06 (2.04)	4.29 (2.03)	0.438
TC [mean (SD)]		3.92 (0.82)	3.89 (0.84)	3.95 (0.80)	0.594
Lipoprotein.A [mean (SD)]		278.94 (33.38)	279.03 (33.67)	278.82 (33.22)	0.966
Age [mean (SD)]		59.77 (11.77)	60.20 (12.15)	59.27 (11.35)	0.591
Progressive (%)	No	153 (81.8)	86 (85.1)	67 (77.9)	0.276
	Yes	34 (18.2)	15 (14.9)	19 (22.1)	
Hypertension (%)	No	89 (47.6)	54 (53.5)	35 (40.7)	0.111
	Yes	98 (52.4)	47 (46.5)	51 (59.3)	
DM (%)	No	90 (48.1)	57 (56.4)	33 (38.4)	0.02
	Yes	97 (51.9)	44 (43.6)	53 (61.6)	
Smoke (%)	No	117 (62.6)	71 (70.3)	46 (53.5)	0.027
	Yes	70 (37.4)	30 (29.7)	40 (46.5)	
Alcohol (%)	No	122 (65.2)	71 (70.3)	51 (59.3)	0.156
	Yes	65 (34.8)	30 (29.7)	35 (40.7)	
AF (%)	No	173 (92.5)	97 (96.0)	76 (88.4)	0.088
	Yes	14 (7.5)	4 (4.0)	10 (11.6)	
Education (%)	High	111 (59.4)	73 (72.3)	38 (44.2)	<0.001
	Low	76 (40.6)	28 (27.7)	48 (55.8)	
CHD (%)	No	150 (80.2)	80 (79.2)	70 (81.4)	0.849
	Yes	37 (19.8)	21 (20.8)	16 (18.6)	
PVD (%)	No	150 (80.2)	80 (79.2)	70 (81.4)	0.849
	Yes	37 (19.8)	21 (20.8)	16 (18.6)	
Gender (%)	Female	88 (47.1)	47 (46.5)	41 (47.7)	0.993
	Male	99 (52.9)	54 (53.5)	45 (52.3)	
Stroke localization (%)	Left	71 (38.0)	31 (30.7)	40 (46.5)	0.038
	Right	116 (62.0)	70 (69.3)	46 (53.5)	
Reperfusion therapy (%)	No	154 (82.4)	83 (82.2)	71 (82.6)	1
	Yes	33 (17.6)	18 (17.8)	15 (17.4)	

NIHSS, National Institutes of Health Stroke Scale; DM, diabetes mellitus; AF, atrial fibrillation; CHD, coronary heart disease; PVD, peripheral vascular disease.

### 3.2 Temporal changes of the patients’ cognitive performance

Among 573 patients with AIS, 61.95% (355) experienced acute cognitive impairment. Of the 355 patients with acute cognitive impairment, 32.39% (115) regained normal cognitive status (MOCA ≥26) six months after AIS. Furthermore, 9.17% (17) of 218 patients with normal baseline cognitive status progressed to PSCI six months after AIS. Based on their MoCA scores, these patients were categorized into two distinct groups: PSCI (consisting of 260 cases) and CN (consisting of 313 cases).

### 3.3 Variable selection for the predictive model of PSCI after ischemic stroke

Through the application of multifactor logistic analysis coupled with bidirectional stepwise regression techniques, the variables that were ultimately incorporated into the regression model encompassed the NIHSS score upon admission, stroke progression, diabetes mellitus status, prior history of atrial fibrillation, HCY levels, smoking habits, alcohol consumption, gender, educational attainment, stroke localization, and Lp-PLA2. The outcomes of this analysis are presented in Table 4. The formulated model can be expressed as follows: PSCI = f (NIHSS, Progressive, HCY, DM, Smoking, Alcohol, AF, Education, Gender, Lp-PLA2, Localization).

**Table 4:** Stepwise logistic regression with bidirectional selection of variables.

Variables	*β*	OR	95%CI	*P*
NIHSS	0.0892	1.093	1.025–1.168	0.0007
Stroke progressive	1.34	3.815	1.75–8.68	0.0001
HCY	0.032	1.033	1.007–1.06	0.0136
DM	0.966	1.58	1.53–4.606	<0.0001
Smoke	1.871	6.495	3.21–13.77	<0.0001
Alcohol	2.253	9.514	4.56–20.86	<0.0001
AF	0.9	2.46	1.05–5.98	0.0414
Education	1.52	4.58	2.62–8.21	<0.0001
Gender	−2.144	0.117	0.051–0.254	<0.0001
Lp.pla2	0.005	1.005	1.0009–1.009	0.016
Stroke localization	−1.67	0.188	0.108–0.319	<0.0001
CHD	−2.05	0.15	0.35–1.05	0.06
LDL.C	−0.38	0.69	0.42–1.04	0.08

NIHSS, National Institutes of Health Stroke Scale; DM, diabetes mellitus; AF, atrial fibrillation; CHD, coronary heart disease.

### 3.4 Establishment and validation of the clinical prediction model for the occurrence of PSCI in patients with acute ischemic stroke

**Figure 2:**
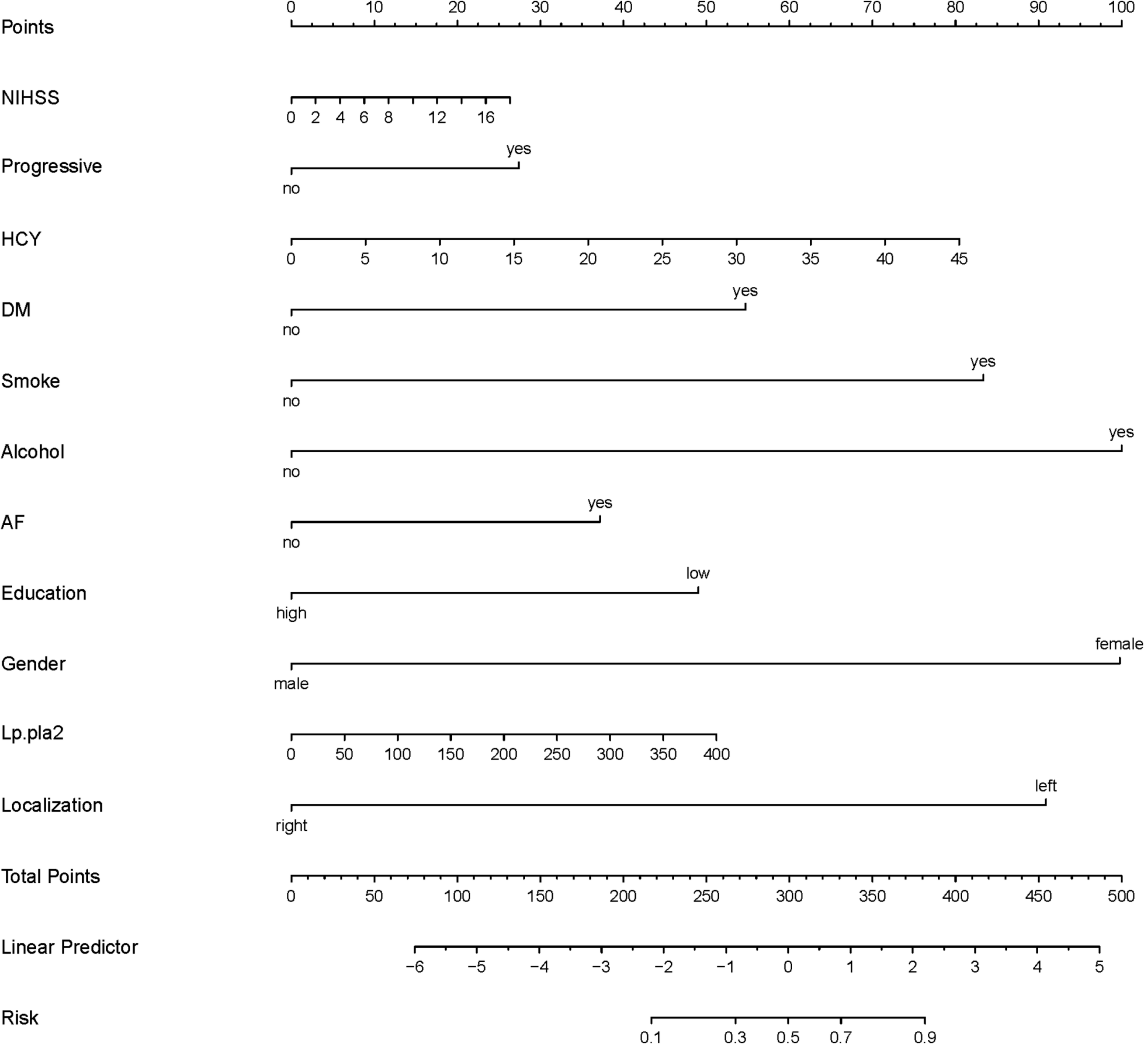
Nomogram (development corhort); The nomogram presents a visual method to calculate the probability of PSCI within 6 months after acute ischemic stroke based on a patient’s admission characteristics. To calculate the probability of PSCI, sum up the points identified on the scale for each of the eleven variables (NIHSS, Progressive, HCY, DM, Smoking, Alcohol, AF, Education, Gender, Lp-PLA2, Stroke localization) to obtain the total points. Draw a vertical line down from the total points scale to the last axis to obtain the corresponding probability of PSCI.

**Figure 3:**
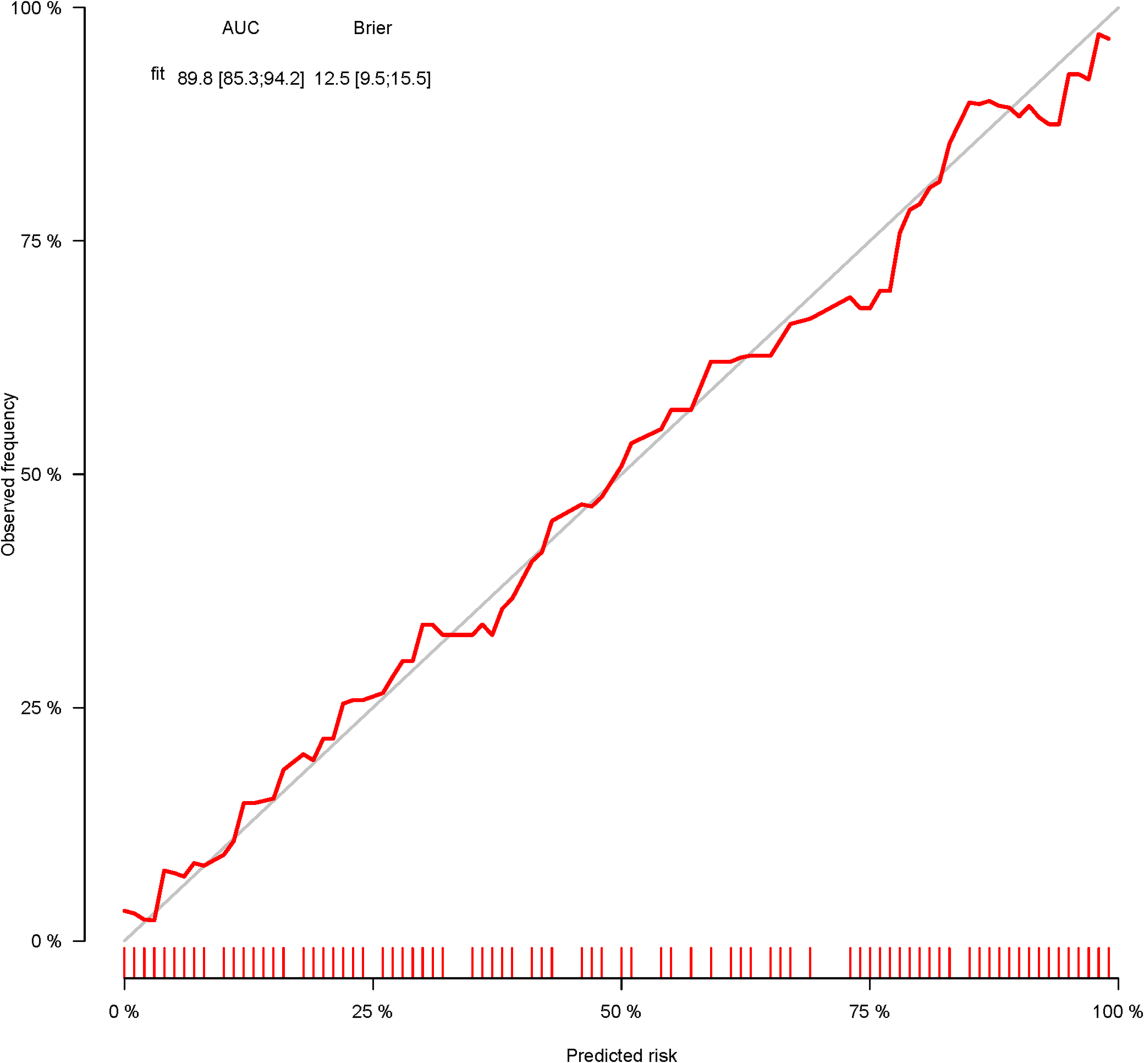
Calibration plot for predicting PSCI in patients with AIS in the development corhort.

**Figure 4:**
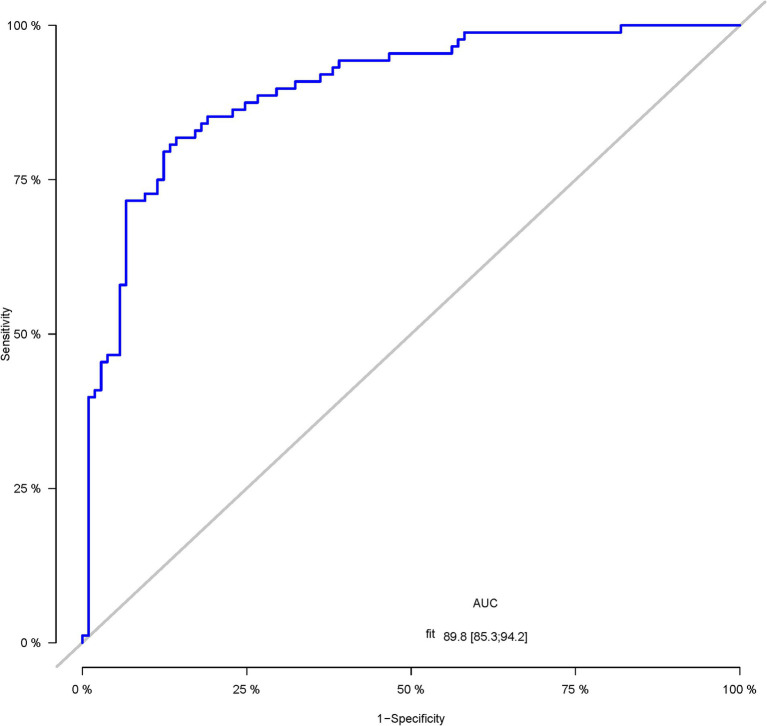
The ROC curve of the nomogram for predicting PSCI in patients with acute ischemic stroke in the development corhort.

**Figure 5:**
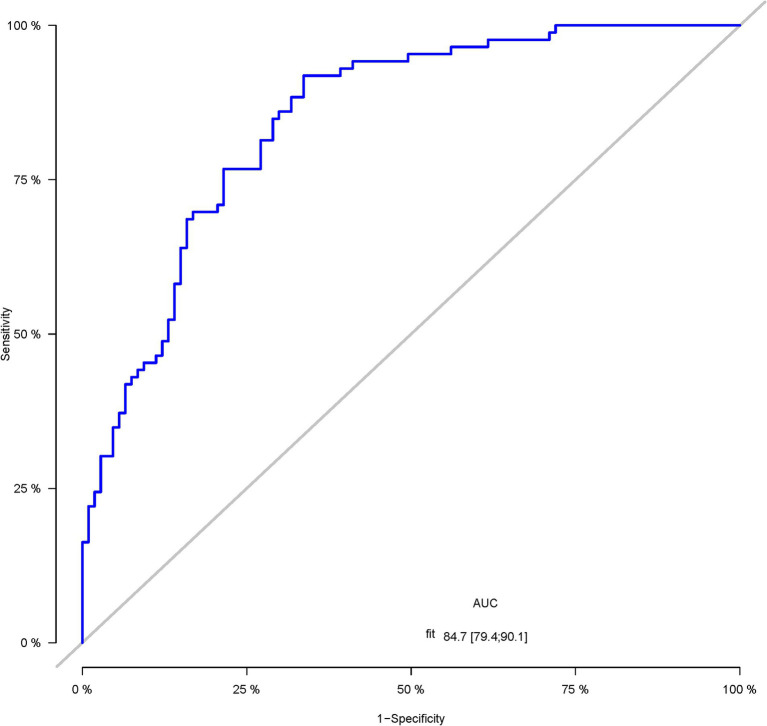
The ROC curve of the nomogram for predicting PSCI in patients with acute ischemic stroke in the internal validation corhort.

**Figure 6:**
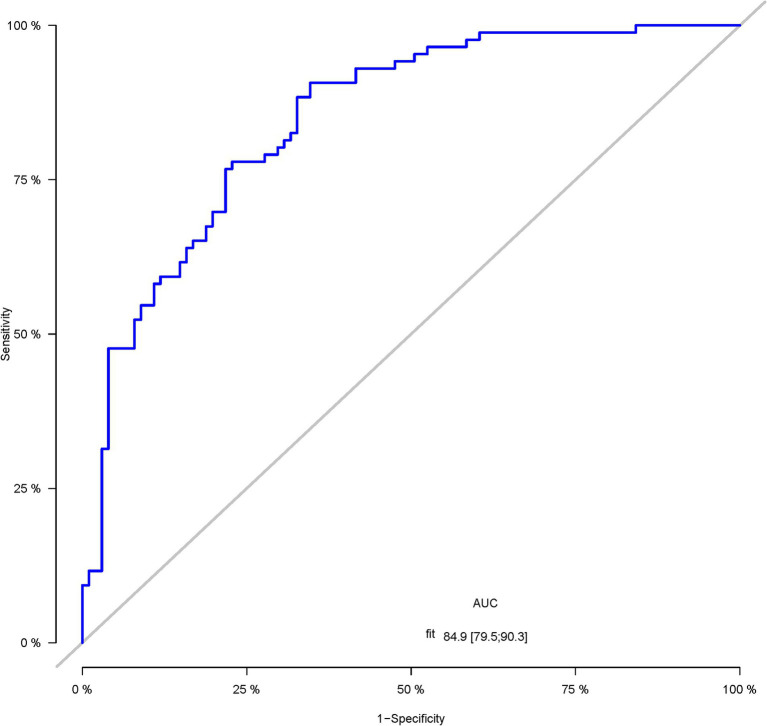
The ROC curve of the nomogram for predicting PSCI in patients with acute ischemic stroke in the external validation corhort.

**Figure 7:**
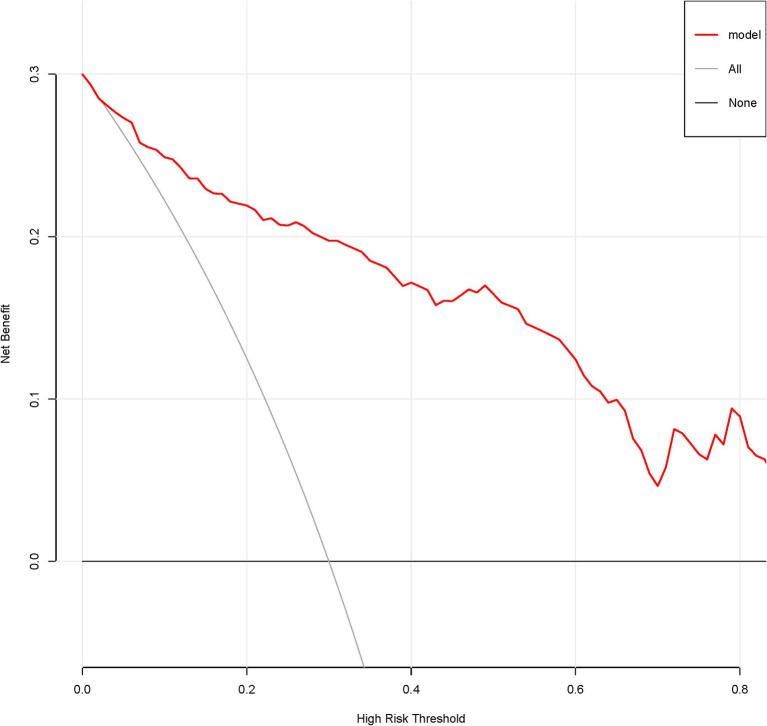
Decision curve of the nomogram predicting PSCI in patients with acute ischemic stroke in the development corhort.

A total of 386 patients with acute ischemic stroke (AIS) admitted to the Stroke Center of Huai’an First People’s Hospital were randomly assigned to a development cohort and an internal validation cohort in a 1:1 ratio. Consequently, the development cohort comprised 193 AIS patients, and the internal validation cohort included 193 AIS patients. An external validation cohort consisting of 187 AIS patients was sourced from the Stroke Center of the Affiliated Huai’an Hospital of Xuzhou Medical University. The predictive framework incorporated eleven predictors: smoking, alcohol consumption, female sex, educational attainment that is low, the NIHSS score upon admission, stroke progression, diabetes mellitus, a history of atrial fibrillation, stroke localization, HCY, and Lp-PLA2. These variables were harnessed to construct a nomogram (depicted in Figure 2). An evaluation of the calibration curve (Figure 3), along with the Hosmer-Lemeshow test for goodness-of-fit, internal validation, and external validation, collectively attests to the model’s robust predictive capabilities. The area under the receiver operating characteristic curve (AUC) for the predictive model in the development cohort amounted to 0.898 (95% CI: 0.853 to 0.942) (Figure 4), accompanied by a Hosmer-Lemeshow chi-squared statistic of 3.2885 and a *p*-value of 0.915. For internal validation, the study enrolled 193 patients with acute ischemic stroke. The precision of the derived model, after 1,000 iterations of bootstrap resampling, stood at 84.7% (95% CI: 79.4 to 90.1%). The AUC of the predictive model in the internal validation cohort was 0.847 (95% CI: 0.794 to 0.901) (Figure 5), with a Hosmer-Lemeshow chi-squared value of 15.04 and a *p*-value of 0.06. The external validation cohort comprised 187 patients with acute ischemic stroke from a different stroke center. The accuracy of the model, based on 1,000 bootstrap resampling repetitions, was 84.9% (95% CI: 79.46 to 90.31%). Furthermore, the AUC of the predictive model in the external validation cohort was 0.849 (95% CI, 0.7946 to 0.9031) (Figure 6), with a Hosmer-Lemeshow chi-squared value of 11.6 and a corresponding *p*-value of 0.17. As illustrated in Figure 7, a discernible trend emerges, where the net benefit of the decision curve analysis exhibits a marked elevation when the threshold probability for PSCI lies between 0.05 and 0.79. This finding contrasts with the “None” and “ALL” approaches, suggesting that the decision curve analysis holds promising clinical utility.

## 4 Discussion

In our investigation, we noted a 45.4% incidence rate of post-stroke cognitive impairment, aligning with previous literature (2, 4, 6, 18). By examining acute ischemic stroke data, we found that PSCI typically manifests within six months following an AIS event. Our results highlighted smoking, alcohol use, female gender, low educational attainment, admission NIHSS score, stroke progression, stroke localization, diabetes, atrial fibrillation history, HCY levels, and Lp-PLA2 as significant predictors of PSCI. These variables were chosen as independent predictors. We subsequently developed and validated a clinical prediction model that accurately forecasts the occurrence of PSCI in AIS patients, enabling the identification of high-risk individuals for timely interventions aimed at mitigating PSCI incidence.

In our study, we identified CHD and low-density LDL-C as potential protective factors against PSCI after acute ischemic stroke. However, this finding contrasts with previous research report (19) that CHD as a risk factor for cognitive impairment after stroke but are less definitive about LDL-C. A comprehensive meta-analysis leveraging individual participant data from four longitudinal studies in the United States, spanning the period from 1971 to 2019, was conducted. This collective examination of cohorts revealed an absence of statistically significant associations between post-stroke LDL-C levels and subsequent cognitive decline among stroke survivors (20). Dong (15) et al. reported that LDL-C had a paradox effect on the risk of PSCI. The inconsistencies between our findings and those of previous studies may stem from several sources. Firstly, differences in study design, including sample selection, sample size, and duration of follow-up, can significantly impact the ability to detect associations between risk factors and outcomes. Secondly, variations in cognitive assessment tools and diagnostic criteria for cognitive impairment can lead to discrepancies in reported results. Thirdly, population-specific factors, such as genetic predispositions, environmental exposures, and healthcare practices, could contribute to variability in the observed associations. Furthermore, the role of potential confounding and modifying factors, such as medications, lifestyle habits, and coexisting medical conditions, must be carefully considered. These factors can interact in complex ways that influence the relationship between risk factors and post-stroke cognitive impairment. Future studies with larger sample sizes, longer durations of follow-up, and more uniform cognitive assessment tools are needed to confirm these findings and elucidate the complex relationships between risk factors and post-stroke cognitive impairment.

Our predictive model has the potential to facilitate early recognition of AIS patients at medium to long-term risk for PSCI. Notably, most of the variables incorporated into our model’s risk score have been previously established as crucial risk factors for PSCI in various studies (1, 17, 21–28). Several existing prediction tools incorporate neuroimaging techniques (1, 3, 27, 28), such as the SIGNAL2 risk score, which includes age, education level less than six years, overall cortical atrophy stage, Fazekas stage, non-cavitary cortical infarction stage, chronic cavities ≥2, and intracranial arterial stenosis (21). Similarly, the CHANGE risk score considers age, education level, cortical atrophy, acute non-cavitary cortical infarction, white matter hyperintensity (WMH), and chronic cavity status (1). The GRECogVASC cognitive risk score comprises NIHSS score at admission ≥7, multiple strokes, corrected Mini-Mental State Examination (MMSE) ≤27, and Fazekas score ≥ 2 (26). Ding et al. also established a PSCI risk model encompassing age, years of education, periventricular hyperintensity grade, diabetes, and the number of acute non-cavitary infarctions (25). Importantly, all these models necessitate neuroimaging data (1, 21, 25, 26). In contrast, our risk score does not rely on neuroimaging, rendering our predictive model simpler and more feasible for implementation in stroke units offering intensive care. However, further testing and validation in diverse AIS patient populations are required.

Our study has inherent limitations. Firstly, the number of PSCI cases was relatively small, limiting our ability to perform further PSCI subtyping due to the sample size constraints and lack of specific diagnostic classifications. Secondly, as this was a six-month study, we could not assess the cognitive function evolution beyond this period, nor fully evaluate the impact of stroke severity on PSCI risk. Thirdly, in our retrospective study, pre-stroke MOCA scores were indeed challenging to ascertain due to the acute nature of stroke and the reliance on existing medical records. These scores were primarily obtained from documented evaluations in the patients’ medical charts, which may have been conducted as part of routine pre-admission assessments or during previous visits for unrelated conditions. However, it is acknowledged that not all patients had a pre-stroke MOCA evaluation recorded in their medical history. In such cases, we relied on reported histories of normal cognition, as documented by the treating physicians or based on patient and family reports. While this approach allowed us to include a larger sample size in our analysis, it does introduce potential bias and limitations to our study. Moreover, the influence of rehabilitation therapy on patients’ neurological function, particularly cognitive function, was not comprehensively examined. These limitations are typical of retrospective studies. Fourthly, the MoCA test, originally designed for mild cognitive impairment detection, is not specific to PSCI and excludes some common cognitive impairments (e.g., post-stroke aphasia, neglect, visual impairment, apraxia, reading, and writing difficulties). However, MoCA remains a widely used bedside screening tool for PSCI. Therefore, AIS patients with severe stroke symptoms unable to complete the MoCA test can be considered a high-risk group for developing PSCI.

In summary, our study offers valuable insights into the frequency of PSCI within six months post-AIS. Our predictive model serves as a reliable screening tool for early identification of high-risk AIS patients prone to developing PSCI. However, multi-center studies with larger AIS cohorts are crucial for further validation of our findings and contributions.

## Data Availability

The raw data supporting the conclusions of this article will be made available by the authors, without undue reservation.
